# The Use of Percutaneous Thermal Sensing Microchips to Measure Body Temperature in Horses during and after Exercise Using Three Different Cool-Down Methods

**DOI:** 10.3390/ani12101267

**Published:** 2022-05-14

**Authors:** Hyungsuk Kang, Rebeka R. Zsoldos, Jazmine E. Skinner, John B. Gaughan, Vincent A. Mellor, Albert Sole-Guitart

**Affiliations:** 1 School of Agriculture and Food Sciences, The University of Queensland, Gatton, QLD 4343, Australia; hyungsuk.kang@uq.net.au (H.K.); r.zsoldos@uq.edu.au (R.R.Z.); jazmine.skinner@usq.edu.au (J.E.S.); j.gaughan@uq.edu.au (J.B.G.); vam103@maths.uq.edu.au (V.A.M.); 2 School of Agriculture and Environment, University of Southern Queensland, Toowoomba, QLD 4350, Australia; 3 School of Veterinary Science, The University of Queensland, Gatton, QLD 4343, Australia

**Keywords:** horse, percutaneous thermal sensing microchip, body temperature, co ol-down method, central venous temperature

## Abstract

**Simple Summary:**

Strenuous exercise can increase the chance of exertional heat illness (EHI) in horses. To alleviate or prevent EHI in horses, aggressive human intervention is critical, as well as frequent monitoring of body temperature. If body temperature could be obtained more accurately, safely, and rapidly post-exercise, it could act as a rapid point-of-care in detecting the early stages of EHI in horses. A percutaneous thermal sensing microchip (PTSM) has the potential to provide an accurate body temperature, more rapidly and safely post-exercise. However, to achieve this, the optimal body site for microchip implantation must be determined. Furthermore, PTSMs have only be assessed over a short period of time post-exercise in horses. This study was designed to investigate the relationship between the core body temperature and muscle temperatures measured by PTSMs during exercise, and post-exercise application of different cool-down methods. Identifying the accuracy and usefulness of PTSMs as a means of monitoring the body temperature of horses after strenuous exercise, may help to detect the early stages of EHI, and determine if aggressive human intervention is required.

**Abstract:**

The frequent monitoring of a horse’s body temperature post strenuous exercise is critical to prevent or alleviate exertional heat illness (EHI) from occurring. Percutaneous thermal sensing microchip (PTSM) technology has the potential to be used as a means of monitoring a horse’s body temperature during and post-exercise. However, the accuracy of the temperature readings obtained, and their relationship to core body temperature are dependent on where they are implanted. This study aimed to document the relationship between core body temperature, and temperature readings obtained using PTSM implanted in different muscles, during exercise and post application of different cool-down methods. PTSMs were implanted into the right pectoral, right gluteal, right splenius muscles, and nuchal ligament. The temperatures were monitored during treadmill exercise, and post application of three different cool-down methods: no water application (W_no_), water application only (W_only_), and water application following scraping (W_scraping_). Central venous temperature (T_CV_) and PTSM temperatures from each region were obtained to investigate the optimal body site for microchip implantation. In this study, PTSM technology provided a practical, safe, and quick means of measuring body temperature in horses. However, its temperature readings varied depending on the implantation site. All muscle temperature readings exhibited strong relationships with T_CV_ (r = 0.85~0.92, *p* < 0.05) after treadmill exercise without human intervention (water application), while the nuchal ligament temperature showed poor relationship with T_CV_. The relationships between T_CV_ and PTSM temperatures became weaker with water application. Overall, however the pectoral muscle temperature measured by PTSM technology had the most constant relationships with T_CV_ and showed the best potential to act as an alternate means of monitoring body temperature in horses for 50 min post-exercise, when there was no human intervention with cold water application.

## 1. Introduction

Horses compete in diverse and physically strenuous equestrian disciplines. These physically challenging pursuits, and the conditions in which they undertake strenuous exercise can cause rapid increases in body temperature [1]. Without appropriate cool-down intervention, this can progress to exertional heat illness (EHI) [2,3]. Although EHI can occur at lower ambient temperatures, and during a short duration of exercise [4], the chances of developing EHI increase with prolonged exercise, especially under hot and humid conditions which limit the physiological thermoregulatory capacity in horses [5,6,7]. Early detection of EHI immediately post-exercise is important as it can rapidly develop into severe illness causing endotoxaemia, neuronal injury, and/or heat stroke [2,3]. The maximum internal body temperature (gastrointestinal tract) of trotters has been documented for up to 34 min post-exercise [8]. Also, in some endurance horses, post-exercise temperature did not return to baseline for 60 min [8]. Monitoring body temperature following exercise can help in identifying the early warning signs of EHI, and therefore assist in the intervention decision-making process. Currently, rectal temperature via a digital thermometer is the most commonly used technique to measure body temperature in horses [2,9,10,11]. However, it is occasionally not practical and/or safe to obtain rectal temperature after exercise. An infrared thermometer could be a practical method to measure the body temperature of horses post-exercise [12,13]. However, this technique may not be reliable due to the poor correlation between thermographic temperature and rectal temperature [12,13]. Furthermore, a recent study found that the distance, angle, ambient radiation, and light can affect the body temperature readings obtained by an infrared thermometer [14]. Another way of monitoring body temperature is the use of percutaneous thermal sensing microchip (PTSM), and the use of these has been investigated in a number of animal species [15,16,17]. This method has been documented to be faster and easier for obtaining body temperature, compared to the placement of the conventional digital thermometer in the rectum [15,16,17]. In a recent study in exercised horses, the temperature of the pectoral and gluteal muscle obtained by PTSMs showed a strong correlation to core body temperature (central venous temperature) during, and immediately after, treadmill exercise [18]. However, PTSMs have not yet been tested during and after cooling horses down post-exercise.

In addition to measuring body temperature to detect early signs of EHI immediately after exercise, implementation of effective cooling techniques for horses with EHI can help to prevent deleterious illness, such as endotoxaemia, neuronal injury, or heat stroke [2,3]. There have been several efforts to find the most effective cool-down method, such as the application of large volumes of cold water over the horses’ entire body, scraping following cold water application, or using fans that spray cool water [19,20,21,22,23]. A previous cool-down study documented that continuous application of tap water was the most effective at cooling down horses when compared with cold water (10.4 ± 0.4 °C) application every 3 min, and with cold water application followed by scraping [19]. However, it may not be an efficient method where there is limited access to tap water, and it requires horses to stand in a specially designed stock which may not be practical or feasible in field conditions. More aggressive intervention with cold water application, such as applying cooler water under shorter intervals, may reduce body temperature more rapidly and, additionally, more efficiently.

If PTSM technology can reliably and accurately measure the body temperature during recovery post-exercise, it could significantly improve the welfare of horses by monitoring and detecting early changes in their body temperature. It may also assist in the decision-making process in determining whether more aggressive intervention is required for horses to relieve severe heat stress in different equestrian disciplines. However, to-date there is no studies comparing core body temperature and PTSM temperatures during recovery post-exercise while cooling the horses. Previous studies comparing scraping versus not scraping of cold water were performed using the pulmonary artery temperature and rectal temperature [19,24]. Therefore, additional information about the effects of different cool-down methods on the body temperatures using PTSM will help to further understand the possibility of using this technology to monitor the body temperature of horse’s post-exercise.

The purpose of the present study was to assess the relationship among central venous temperature (the gold standard in this study), rectal temperature, and other body temperatures measured by PTSMs during application of different cool-down methods (no water application (W_no_), water application only (W_only_), and water application following scraping (W_scraping_)) post-exercise. We hypothesized that the pectoral muscle temperature measured by PTSM would have a strong relationship to central venous temperature during cooling down post-exercise.

## 2. Materials and Methods

### 2.1. Horses

Four Standardbred geldings and one Thoroughbred gelding, aged 9.6 ± 2.9 years and weighing 518.3 ± 16.2 kg, were used in this study. The horses were housed in individual yards for the duration of the experimental period. They were fed 1.5% BW/day of lucerne hay fed out twice a day, and water was available *ad libitum*. Lameness was examined by an equine surgeon specialist (A.S.G.) before and during the study, and the horses were deemed to be clinically healthy. The study was approved by the Animal Ethics Committees (AEC) of The University of Queensland (Approval no. SVS/341/20).

### 2.2. Preparation for Body Temperature Measurements

#### 2.2.1. Implantation of Percutaneous Thermal Sensing Microchip (PTSM)

Each horse was implanted with four percutaneous thermal sensing microchips (LifeChip^®^ with Bio-thermo^TM^; Destron Fearing^TM^; Dallas, TX, USA). These were implanted into the nuchal ligament, the right splenius muscle, the right gluteal muscle, and the right pectoral muscle (size of the microchip; 2.12 ± 0.10 mm in diameter,13.00 ± 0.40 mm in length, and weighing 0.11 ± 0.03 g) [25]. The body temperatures in the right pectoral muscle (T_PM_), the gluteal muscle (T_GM_), the right splenius muscle (T_SM_), and the nuchal ligament (T_NL_) were measured using two microchip scanners (GPR+; Destron Fearing^TM^; Dallas, TX, USA).

Each body site for implanting PTSM was measured using set parameters to ensure uniformity of the position across the horses. The set parameter for the nuchal ligament PTSM was halfway between the poll and the withers; for the right splenius muscle PTSM it was halfway between the poll and the middle of the scapular spine; for the right gluteal muscle PTSM it was in the intersection halfway between the tail head and the right tuber coxae; and for the right pectoral muscle PTSM it was in the middle of the right cranial pectoral muscle.

Before implantation of PTSMs, the horses were standing in a stock and sedated using xylazine (0.3–0.4 mg/kg body weight). The hair on each implantation site was clipped, and the skin was surgically disinfected using betadine and alcohol. Three ml of local anesthetic (Lignocaine Hydrochloride 20 mg/mL) was injected into the sites subcutaneously. Each microchip was implanted perpendicular to the skin to the maximum depth allowed by the pre-sterilized 12-gauge needle assembly containing the temperature sensor.

The microchips in the splenius muscle, the gluteal muscle, and the pectoral muscle, were implanted during our previous study (March 2019) [18]. The microchip into the nuchal ligament was implanted into the same horses two weeks before the commencement of this study (October 2020). The implanted PTSMs were not removed after the study, and the horses remained as part of the research herd at the University of Queensland.

#### 2.2.2. Rectal Temperature (T_R_) Probe Placement

A temperature data logger (HOBO Pro v2; U23-002; Onset Computer Corporation; Bourne, MA, USA) with a 184 cm thermistor lead attached was used to obtain rectal temperature (T_R_) during treadmill exercise and recovery post-exercise. To secure the thermistor in the rectum, it was fed through a sterilized 57 cm pipette (Equine universal AI pipette; Minitüb GmbH; Tiefenbach, Germany) and inserted 40 to 50 cm deep into the rectum. The data logger was then secured to the tail using vet wrap.

#### 2.2.3. Central Venous Temperature (T_CV_) Probe Insertion

To obtain T_CV_, a type T flexible implantable thermocouple (Physitemp Instrument; Clifton, NJ, USA) was used. Hair on a small area in the cranial third of the left jugular groove was clipped and clinically disinfected, and the thermocouple was inserted with subcutaneous lignocaine (anaesthetic) into the left jugular vein through the lumen of a 14 Gauge 8 cm intravenous catheter (AngiocathTM; Becton-Dickinson and company; Franklin Lakes, NJ, USA). The thermocouple was inserted 80 cm deep into the jugular vein through the IV catheter toward the thorax. Once the thermocouple was introduced, the head of the thermocouple was secured on the skin using sutures. The central venous temperature measured by the thermocouple was displayed on a monitor (Thermalert model TH-8; Physitemp Instrument; Clifton, NJ, USA).

### 2.3. Cool-down Methods

**Figure 1:**
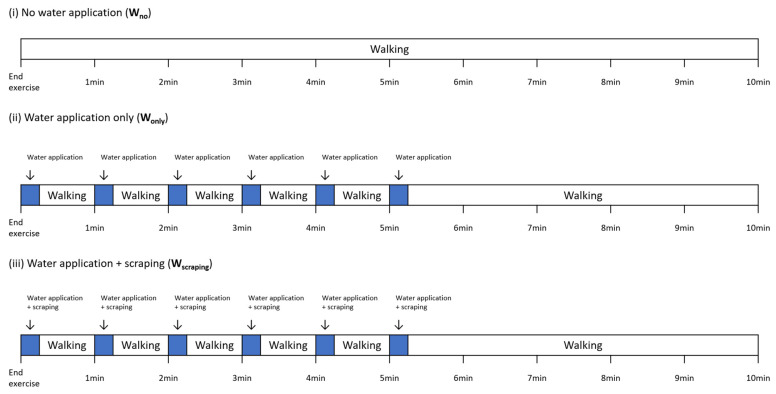
The three cool-down methods applied post treadmill exercise. (**i**) no water application (W_no_), (**ii**) water application only (W_only_), and (**iii**) water application following scraping (W_scraping_). Each cool-down method was applied during the first 10 min post treadmill exercise.

To assess the relationships among the body temperatures obtained during the application of different cool-down methods post-exercise, three cool-down methods were used (Figure 1): 

The first cool-down method, no water application (W_no_), consisted of continuously hand walking the horses for 10 min in an undercover area next to the treadmill room (Figure 1).

The second cool-down method, water application only (W_only_), consisted of six repeats of cold water (6 °C) application with one min intervals for the first six minutes, and followed by continuously hand walking for the next four minutes (Figure 1). The cold water was prepared in advance using ice and tap water until the water temperature was approximately 6 °C. Thirty liters of cold water were evenly applied to the left and right sides of the horses from neck to tail using five-liters buckets. During the cold-water application, the horses were stood still. In between the repeats of cold-water application, the horses were hand walked until the next cold-water application resumed (Figure 1).

The third cool-down method, water application following scraping (W_scraping_), also consisted of six repeats of the cold-water application, but the water was scraped off after each water application. After the six repeats of cold-water application and scraping, the horses were continuously hand walking for the next four minutes. The horses were also stood still during the cold-water application, and during the scraping off, of the water (Figure 1).

### 2.4. Conditioning Period (before Data Collection)

Before commencement of the current study, the horses were turned out in a large paddock at The University of Queensland, Gatton Campus for research and teaching purposes without routine training. In order to familiarize the horses with the data collection procedure, the horses had a three-week conditioning period (week 1–3). During this period, the horses were exercised using a horse walker (Evolution series land walkers; Irongate Australia, SA, Australia) three days a week under an incremental exercise program. The horses exercised evenly clockwise and counterclockwise. Details of the exercise program during the conditioning period are shown in Table A1 in Appendix A. Also, to familiarize the horses with the cooling regimes, the horses were cooled down with tap water using buckets and two scrapers after the exercise. 

### 2.5. Exercise and Cool-Down Program

#### 2.5.1. Treadmill Exercise Program

**Figure 2:**
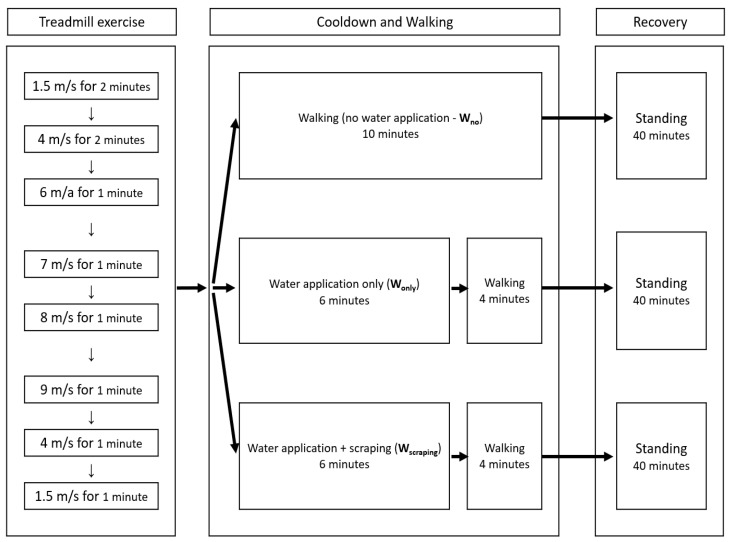
Exercise and cool-down program during the data collection period.

After the conditioning period (weeks 1–3), the horses had a three-week experimental data collection period on the treadmill (weeks 4–6). The horses were introduced to the treadmill room and treadmill machine (Veterinary Pit Model 980; Classic Treadmills Australia PTY Ltd., Kenilworth, QLD, Australia) prior to the data collection period. The horses were exercised on the treadmill once day a week to collect temperature data, and exercised on the horse walker two days a week to maintain consistent physical conditioning. The treadmill exercise was set for 10 min with a predetermined exercise program (Figure 2). The treadmill room had a centrally controlled air-conditioning system, and two wall-mounted fans provided 5 m/s wind speed while horses were exercising. Details of the horse walker program during the data collection period are shown in Table A1 in Appendix A.

#### 2.5.2. Application of the Three Cool-Down Methods (Cool-Down/Walking Phase)

All five horses exercised on the treadmill three times, once a week for three consecutive weeks (weeks 4–6). This was followed by one of the three cool-down methods (details in 2.3. three cool-down methods). Horses were randomly assigned to one of the three cool-down methods, and each horse completed each of the cool-down methods in a cross-over design (W_no_, W_only_, and W_scraping_) over the data collection period. 

#### 2.5.3. Recovery

After the 10-min cool-down/walking phase, the horses then completed a stationary 40 min recovery phase, in the same undercover area where the cool-down/walking period was conducted. Ad libitum access to water was available during this phase, and the horses were held by a handler.

The undercover area, where the cool-down/walking phase and recovery phase were conducted, had natural ventilation and did not have air conditioning. The entire exercise, cool-down/walking, and recovery phases during the data collection period are shown in Figure 2.

### 2.6. Data Acquisition

All the PTSM temperatures were obtained at the same time, at 30 s intervals during 10 min of the treadmill exercise, at 1 min intervals during 10 min of the cool-down/walking phase, and at 5 min intervals during 40 min of the recovery phase. 

The central venous temperature was obtained at 1 s intervals and T_R_ was obtained at 30 s intervals. All temperature data was matched to the time points of the microchip temperature measurements.

Ambient temperature and relative humidity in the treadmill room were measured once at the beginning of each treadmill exercise by a handheld heat stress tracker (4400 Heat Stress Tracker; Kestrel Meters, PA, USA). Ambient temperature and relative humidity in the undercover area next to the treadmill room, were measured once at the beginning of the recovery phase by the handheld heat stress tracker.

### 2.7. Statistical Analysis

The obtained data (T_CV_, T_PM_, T_NL_, T_GM_, T_SM_, and T_R_) was pooled and divided by the phases and by the cool-down methods. The body temperatures measured by PTSM and T_R_ were paired with T_CV_ (T_CV_/T_PM_ pair, T_CV_/T_GM_ pair, T_CV_/T_SM_ pair, T_CV_/T_NL_ pair, T_CV_/T_R_ pair) to be calculated by paired comparison analysis.

For the paired comparison analysis of the body temperature pairs, the repeated measures correlation coefficients analysis and the Bland–Altman test were used. The repeated measures correlation coefficient was used for the associative relationships of the temperature pairs. The coefficients were determined based on previously used categories (|r| = 1: Perfect correlation, 0.9 > |r| > 0.7: Strong correlation, 0.6 > |r| > 0.4: Moderate correlation, 0.3 > |r| > 0.1: Weak correlation, and r = 0: Zero correlation) [26]. The Bland–Altman test was used to assess the level of agreement of the body temperature pairs.

Before comparing the effect of the cool-down methods on the body temperatures, each component was fitted first by a linear mixed-effects (LME) model, and then analyzed by Analysis of Variances (ANOVA) and estimated marginal means of linear trend (emtrends). The significance level was set at *p* = 0.05. For fitting each component by the LME model, week (the three consecutive data collection weeks, week 4–6) and horse (the five horses) were set as random effects, with an autocorrelation correlation structure and phase (cool-down/walking phase and recovery phase), treatment (the three different cool-down methods), and time (body temperature measurement time after the treadmill exercise) were set as fixed effects. To display the differences by phase and treatment, letters were used for grouping each component. The grouping is respective to body temperature, as each body temperature measurement was analyzed individually.

All statistical analyses were conducted using R 4.1.2 [27] using packages ‘rmcorr’ [28], ‘blandr’ [29], ‘nlme’ [30], and ‘emmeans’ [31].

## 3. Results

### 3.1. Air Temperature and Relative Humidity

The average air temperature was 26.06 ± 2.55 °C, and the relative humidity was 48.88 ± 10.29% during the conditioning periods. The average air temperature in the treadmill room was 27.22 ± 1.26 °C, and relative humidity was 49.89 ± 7.16%. The average air temperature in the undercover area was 30.81 ± 2.10 °C, and relative humidity was 47.18 ± 7.66%.

### 3.2. Treadmill Exercise

**Figure 3:**
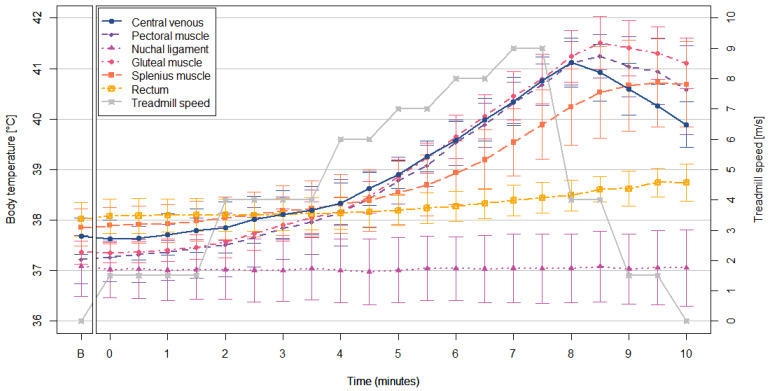
The PTSM temperatures and treadmill speed during the treadmill exercise. Abbreviations: B = before treadmill exercise.

The body temperature changes during the treadmill exercise are shown in Figure 3. During the treadmill exercise, the repeated measures correlation coefficient analysis indicated that the T_CV_/T_PM_ pair had a very strong correlation (r_rm_ = 0.98, *p* < 0.01), which was the strongest among the temperature pairs. It was followed by T_CV_/T_GM_ pair (r_rm_ = 0.97, *p* < 0.01), T_CV_/T_SM_ pair (r_rm_ = 0.87, *p* < 0.01), and T_CV_/T_R_ pair (r_rm_ = 0.76, *p* < 0.05). The analysis did not detect a significant correlation between T_NL_ and T_CV_ (*p* = 0.66), while T_NL_ had a strong correlation with T_R_ (r_rm_ = 0.81, *p* < 0.01).

### 3.3. Paired Comparison Analysis between the Body Temperatures after the Treadmill Exercise

**Figure 4:**
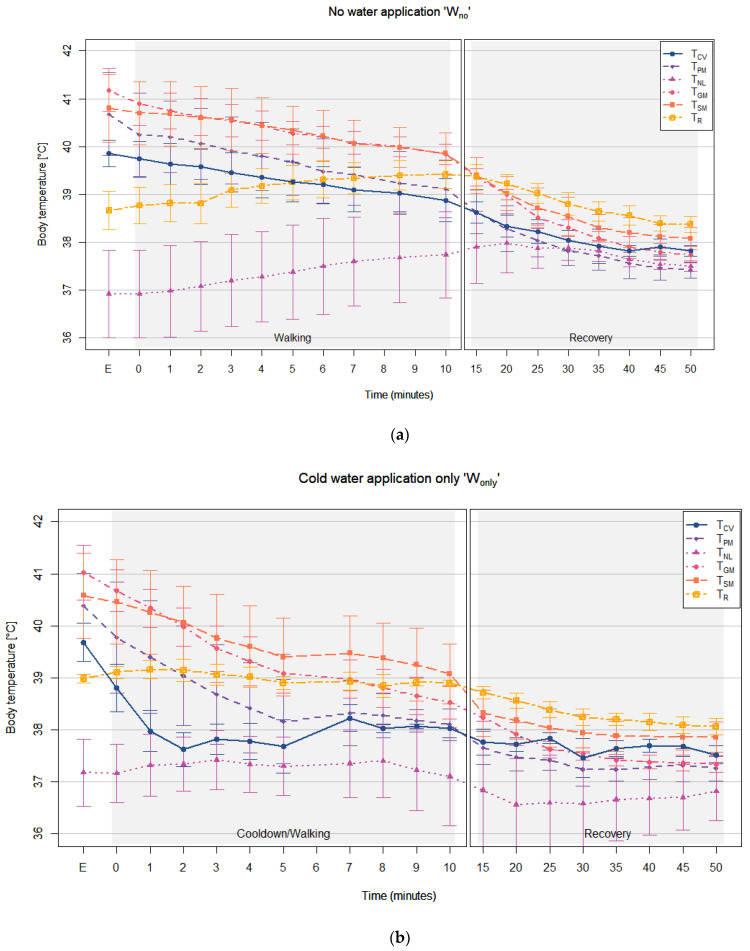

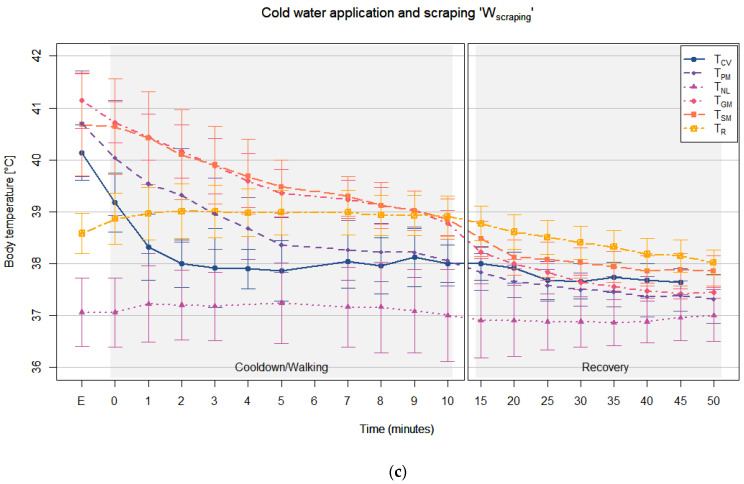
The PTSM temperatures post-exercise during each of the three cool-down methods, (**a**) no water application (continuously walking), (**b**) cold water application only, and (**c**) cold water application and scraping. Abbreviations: E = end of treadmill exercise, T_CV_ = central venous temperature, T_PM_ = pectoral muscle temperature, T_NL_ = nuchal ligament temperature, T_GM_ = gluteal muscle temperature, T_SM_ = splenius muscle temperature, T_R_ = rectal temperature.

The body temperatures detected during the cool-down/walking phase post treadmill exercise, during each of the cool-down methods, are shown in Figure 4a–c. Descriptive statistics for the body temperature data collected at each PTSM site are shown in Table A2 in the Appendix A.

#### 3.3.1. Repeated Measures Correlation Coefficients

Results of the repeated measures correlation coefficient analysis of the body temperature pairs, by phase, and by cool-down methods, are shown in Table 1.

When there was no human intervention (W_no_), all three muscle temperatures had strong positive correlations with T_CV_ without cold water application (*p* < 0.01) during both the cool-down/walking phase and recovery phase. Conversely, T_NL_ and T_R_ had strong, but negative correlations with T_CV_ during the cool-down/walking phase, without cold water application (W_no_). This can be seen in Figure 4a, where T_CV_ was decreasing while T_NL_ and T_R_ were increasing even after the end of treadmill exercise. These negative correlations changed to positive correlations during the recovery phase, as both T_NL_ and T_R_ began to decrease during this phase. However, unlike T_NL_, which had only a moderate correlation with T_CV_ (r_rm_ = 0.47, *p* < 0.01), T_R_ had a strong correlation (r_rm_ = 0.84, *p* < 0.01) with T_CV_, which is not much different to T_PM_ and T_GM_ during the recovery phase. The nuchal ligament temperature had a strong positive correlation with T_R_ (r_rm_ = 0.78, *p* < 0.01) during the cool-down/walking phase with W_no_, but no other correlations were detected between T_NL_ and T_R_.

With cold water application, both W_only_ and W_scraping_, the correlations between T_CV_ and the muscle temperatures were weaker during the cool-down/walking phase than W_no_, and this remained until the recovery phase. Although the correlations were weaker during both phases when compared to W_no_, the muscles temperatures and T_R_ had stronger correlations with T_CV_ during the recovery phase with W_scraping_ than with W_only_. Among the body temperatures, T_PM_ and T_GM_ had better correlations with T_CV_ during the cool-down/walking phase with W_only_ and W_scraping_ than the other body temperatures. However, during the recovery phase, T_GM_ had no significant correlation with T_CV_ using W_only_, while T_PM_ had significant correlations in both cool-down methods. During this recovery phase, the correlations in T_CV_/T_SM_ and T_CV_/T_R_ pairs were similar to T_CV_/T_PM_ pair with both W_only_ and W_scraping_.

#### 3.3.2. Differences

Results of the Bland–Altman analysis of the body temperature pairs by phase, and by cool-down methods, are shown in Table 2.

Without cold water application (W_no_), T_R_ had the least difference (bias) to T_CV_ during the cool-down/walking phase and it was followed by T_PM_. However, during the recovery phase, T_PM_ had smallest difference to T_CV_, which was followed by T_GM_ and T_NL_, while T_R_ had a larger difference to T_CV_, also seen in the bigger gap during the recovery phase in Figure 4a.

With cold water application (W_only_ and W_scraping_), T_PM_ had the smallest difference to T_CV_ during the cool-down/walking phase, as also shown in Figure 4b,c. The T_PM_ cooled down quicker than the other muscle temperatures following a similar pattern as T_CV_. However, during the recovery phase, T_GM_ showed the smallest differences from T_CV_ in both cool-down methods (W_only_ and W_scraping_), followed by T_PM_. The rectal temperature and T_NL_ remained larger different to T_CV_ than the muscle temperatures during the recovery phase with cold water application (W_only_ and W_scraping_).

The limits of agreement (LoA) ranges between T_CV_ and the muscle temperatures were narrower without cold water application (W_no_) than using cooling methods (W_only_ and W_scraping_). The bias and LoA ranges of the body temperature pairs varied depending on the cool-down method, and the PTSM implantation sites. Furthermore, the wide LoA ranges may infer that those body temperatures measured via PTSM, and T_R_ are not equivalent to T_CV_ during the cool-down/walking phase, and recovery phase post-exercise.

### 3.4. Comparison of the Cool-Down Methods and Body Site Temperatures

Using the three-way ANOVA analysis (Table A3 in Appendix A), significant three-way interactions were found only on T_NL_ (*F* (2, 252) = 7.46, *p* < 0.01) and T_R_ (*F* (2, 253) = 15.96, *p* < 0.01). However, the results of the two-way and/or one-way ANOVA indicated that each body temperature was affected differently by the different combinations of the variables (Table A3). Therefore, each variable was also used for the further analysis of body temperature changes during each of the cool-down methods, using ‘emtrends’ analysis as the variables.

The results of the emtrends analysis, are shown in Table A4 in Appendix A.

Without cold water application (W_no_), all the muscle temperatures showed similar temperature changing trends to T_CV_ during the cool-down/walking phase, while T_NL_ and T_R_ did not, as it was also shown by the negative strong correlation coefficients in T_CV_/T_NL_ and T_CV_/T_R_ pairs. However, the analysis indicated that during the recovery phase with W_no_, T_PM_, T_NL_, and T_R_ had similar temperature changing trends to T_CV_, but T_GM_ and T_SM_ did not.

With cold water application (W_only_ and W_scrpaing_), all muscle temperatures also showed similar temperature changing trends to T_CV_ during the cool-down/walking phase and T_NL_ and T_R_ did not, as similar with W_no_. During the recovery phase, T_PM_ and T_SM_ had similar temperature changing trends to T_CV_, but T_GM_. As also shown with W_no_, T_NL_ and T_R_ had similar temperature changing trends to T_CV_ during the recovery phase with cold water application.

The results indicated that T_CV_ and the muscle temperatures, without cold water application (W_no_), and with cold water application (both W_only_ and W_scraping_) were cooled down immediately post treadmill exercise (during cool-down phase in Table A4). The cooling rates of the body temperatures during the recovery phase were significantly decreased, and there was still no significant cooling effect detected for the cold-water application. However, the significant cooling effects of cold-water application, in both W_only_ and W_scraping_, were found in T_NL_ and T_R_, when compared to W_no_, immediately post-exercise, where both body temperatures continuously increased (positive values) without applying cold water. The rectal temperature also increased when the applied cold water was scraped off, but the increasing trend was significantly lower than W_no_. The cooling rates of T_R_ increased during the recovery phase, while T_NL_ barely changed even during the treadmill exercise (Figure 4).

### 3.5. T_CV_ Changes during the Cool-Down Phase

**Figure 5:**
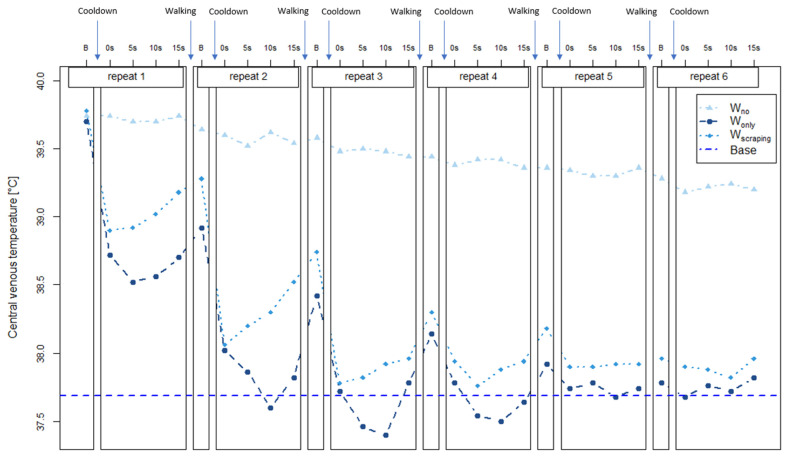
Central venous temperature changes before and immediately after (0–15 s) cold water application during the cool-down phase. Each repeat of cold water application (repeat 1~6) consisted of five-time points, before cold water application (B), immediately after cold water application (0 s), and 5, 10, and 15 s after the time point (5–15 s). In between the repeats, the horses were walked in an undercover area until the next repeat resumed. Abbreviations: W_no_ = no water application (continuously walking), W_only_ = water application, W_scraping_ = water application following scraping, Base = average temperature before treadmill exercise.

Although there was no significant difference found between each of the three cool-down methods (Table A4), it seems that the application of cold water provided a more instant cooling effect, while the T_CV_ was gradually decreased without the cold-water application (W_no_) (First 3 min in Figure 4). To observe the details of T_CV_ changes during the application of cold water, T_CV_ during the cool-down phase (first 6 min immediately post-exercise) is presented in Figure 5. The average T_CV_ before the first cold-water application (Figure 5, ‘B’ in repeat 1) was 39.74 °C for W_no_, 39.70 °C for W_only_, and 39.78 °C for W_scraping_, respectively. There were temperature drops in T_CV_ as soon as cold water was applied post-exercise (both W_only_ and W_scraping_), while there were no changes without the application of cold water. Both water application methods were more effective in cooling down than W_no_. However, T_CV_ with W_scraping_, body temperature began to increase immediately, while T_CV_ with W_only_ (where the cold water remained on the body), showed a further cooling effect before increasing. During the cold-water application of horses, the body temperature of T_CV_ with W_only_ was lower than T_CV_ with W_scraping_. During this period, T_CV_ was 1.88 °C lower with W_only_ and 1.82 °C lower with W_scraping_. While T_CV_ with the cool-down methods was maintained after the fifth application of cold water, T_CV_ with W_no_ continuously decreased. Body temperature was only reduced by 0.54 °C with W_no_. However, due to the consistency of the slow cooling down rate, the emtrends analysis found no significant differences in T_CV_ temperature compared to the other two cool-down methods.

## 4. Discussion

Rectal temperature via a digital thermometer is the most commonly used procedure to monitor the body temperature of horses across a range of equestrian disciplines [32,33,34,35]. While the majority of horses tolerate the placement of a rectal thermometer, and while it is considered a less invasive method than implanting a microchip, there will be occasions where it may not be safe to obtain rectal temperature post-exercise, particularly post competition when horses are thermally challenged [3,36]. Furthermore, as demonstrated in the current study, using rectal temperature may not be the most accurate approach to monitor a horse’s body temperature post-exercise. The rectal temperature measurements during, and immediately after treadmill exercise did not accurately represent the horses’ core body temperature measurements, and therefore the thermoregulation status of the horses’ compared to the temperature measurements obtained from the PTSM, especially the pectoral PTSM [4,13,37]. Previous studies have reported a lower T_R_ compared to T_CV_ after exercise and a lag on the increase of T_R_ compared T_CV_ post-exercise [1,38,39]. The current study also showed similar results, however the differences between T_CV_ and T_R_ in this study were a bit larger than in previous studies at the maximum speed during treadmill exercise. These differences could be related to the different system, depth obtaining the rectal temperature, and exercise protocol [40]. Our current study measured T_R_ approximately 40 to 50 cm deep in the rectum, while other studies measured it at 25 to 30 cm deep in the rectum [1,38,39]. In addition, our treadmill exercise protocol was different than previous studies measuring body temperatures [1,39]. The current study was designed to increase the central venous temperature up to 41 °C compared to previous studies, which were of a different exercise speed and duration and were designed to increase the pulmonary arterial temperature up to 43 °C. The continuous monitoring of body temperature immediately post-exercise is crucial in determining whether a more aggressive intervention to cool horses is required [36,37,41]. However, as rectal temperature did not show a good relationship to core body temperature post-exercise, so the way in which a horse’s body temperature is monitored should be further considered.

There have been efforts to evaluate body temperature using an infrared thermal camera as a safer and more rapid method [42,43]. While it has been demonstrated to be a practical method to use, there is high variability depending on the body site measured, whether the temperature is assessed indoor (or undercover) versus outdoors, and it has not been compared with core body temperature [42,43]. In the current study, PTSM technology was used as an alternate option for monitoring the body temperature of exercised horses. Even though the implantation of standard identification microchips is routine and has been reported to be a stress-free procedure [44] However, the microchipping procedure itself is invasive [45,46]. Once the microchip is implanted, however it can be advantageous by providing a practical, quick, and non-invasive environment for measuring the body temperature of horses before and after exercise, as it requires no animal contact, and takes only a few seconds to obtain the body temperature [15,18,47]. It is crucial to treat heat stressed horses straight away to detect any potential problems, even if they are not currently showing any signs of heat stress. Furthermore, continuously monitoring the temperature of horses is important to observe whether they return to ‘normal’ which is critical to avoid long term irreversible damage [8,48]. Percutaneous thermal sensing microchip technology could easily be used as a screening tool prior to competition to detect those horses at higher risk of developing heat stress. Furthermore, ultrasound examination three months after microchipping detected no migration or foreign body reaction [18], similar to previous reports [44,46,49,50,51,52,53].

Even though PTSM technology is practical, as shown in this study it was not always useful as its accuracy is dependent on where it is implanted. The nuchal ligament temperature showed a very poor relationship to central venous temperature, during and after treadmill exercise. Among the body sites where PTSM was implanted, the pectoral muscle had a strong relationship to central venous temperature post-exercise when there was no water application. The pectoral muscle also had a very strong correlation with T_CV_ during treadmill exercise, and immediately after the exercise [18]. However, the body temperatures obtained from the different sites had different correlations, depending on the phase when the temperature was acquired, and the cool-down method applied. Furthermore, rectal temperature lagged behind central venous temperature, during and after the treadmill exercise, which was also documented in a previous cool-down study [1]. This may be because the body temperatures at the different body sites are influenced by multi factors, such as redistribution of blood flow to dissipate body heat from core to skin during exercise [54,55,56,57], body mass and the surface ratio [6], body fat composition [58], different ages [59,60], or the exercise intensity of each body site [43,56,61,62,63]. Furthermore, interindividual variations could affect the results as well. The broad body temperature variations were recently documented amongst individuals responding to exercise [8]. The current study also showed interindividual variations, but T_PM_ had a slightly larger variation amongst individuals than the other body temperatures during the cooldown/walking phase, particularly during the first 6 min post-exercise. Even though T_PM_ had better relationships with T_CV_ than the other body temperatures immediately post-exercise, it is recommended that a larger number of horses be utilized in future studies to observe the relationship between T_PM_ and T_CV_.

In the current study, three cool-down methods were used to observe their effects on body temperature at each of the PTSM implantation sites. Even though the statistical analysis showed no significant differences between the cool-down methods on T_CV_ during the first 10 min post-exercise, the application of cold water (6 °C) with 1 min intervals between reapplication resulted in a faster cooling effect during the first two minutes, than just walking without the application of cold water. Furthermore, although statistically not different, the cold-water application itself exhibited a slightly further cooling effect on the central venous temperature than scraping the cold water from the body. This has also been documented in a similar previous study [19] suggesting that constant water application is more effective in cooling horses than the use of scraping. The different cooling effects observed may be attributable to heat transfer by conduction from the skin to the cold water, which is more effective at dissipating body heat than evaporation from sweat after scraping. At this current stage, cold water application may be a good option to adapt broadly at both large and small racetracks, or other equestrian disciplines. There have been several efforts to evaluate better cool-down methods for horses [19,20,23,64,65] and it has already been documented that continuously being exposed to cold water, such as immersion or continual hosing of cold water (shower), has the strongest and most immediate cooling effect in humans [66,67,68] and horses [19].

The main limitation of this study was that it was conducted in a controlled indoor setting in only a few horses, that were not physically active in racing or other competitions. Future research needs to be conducted in a larger number of trained horses, in outdoor field conditions, frequent measurement of climate conditions, and under environmental conditions of heat and humidity.

## 5. Conclusions

The results of this study found that the rectal temperature of horses did not accurately represent their core body temperature post-exercise, which is important in detecting the early stages of EHI. The use of percutaneous thermal sensing microchip (PTSM) technology showed good potential to act as an alternative option to continuously monitor the body temperature of horse’s post-exercise. Among the PTSM implantation sites, the pectoral muscle showed the most promising potential to measure temperature when the horses were continuously walking post-exercise without cold water application. However, further research is warranted, as the relationships with the central venous temperature were weakened when cold water was applied post-exercise. Additional investigations using the same technique in horses exercising under hot and humid conditions, is necessary to adapt this technology broadly to screen the body temperature of exercised horses at racetracks and other equestrian disciplines.

## Figures and Tables

**Table 1:** Repeated measures correlation coefficients (r_rm_) between central venous temperature, and the other body temperatures during cool-down/walking phase, and during recovery phase by cool-down method.

	W_no_		W_only_		W_scraping_	
	r_rm_	df	r_rm_	df	r_rm_	df
T_CV_/T_PM_	0.93 **	44	0.40 **	40	0.40 **	44
T_CV_/T_NL_	−0.90 **	44	−0.16 ^n.s.^	40	−0.08 ^n.s.^	43
T_CV_/T_GM_	0.90 **	44	0.42 **	40	0.38 **	44
T_CV_/T_SM_	0.86 **	44	0.34 *	40	0.36 *	44
T_CV_/T_R_	−0.72 **	44	0.09 ^n.s.^	40	−0.35 *	44
T_CV_/T_PM_	0.88 **	34	0.36 *	34	0.61 **	33
T_CV_/T_NL_	0.47 **	34	−0.39 *	34	0.25 ^n.s.^	33
T_CV_/T_GM_	0.85 **	34	0.24 ^n.s.^	34	0.63 **	33
T_CV_/T_SM_	0.89 **	34	0.40 *	33	0.63 **	33
T_CV_/T_R_	0.84 **	34	0.35 *	34	0.54 **	33

Abbreviations: W_no_ = no water application (continuously walking), W_only_ = cold water application only, W_scraping_ = cold water application and scraping, df = degree of freedom, T_CV_ = central venous temperature, T_PM_ = pectoral muscle temperature, T_NL_ = nuchal ligament temperature, T_GM_ = gluteal muscle temperature, T_SM_ = splenius muscle temperature, T_R_ = rectal temperature. *p*-values: ** *p* < 0.01, * *p* < 0.05, ^n.s.^ *p* ≥ 0.05.

**Table 2:** Mean differences (bias) and limits of agreement range (LoA) between the central venous temperature, and the other body temperatures after treadmill exercise by cool-down method.

	W_no_			W_only_			W_scraping_		
	n	bias	LoA	n	bias	LoA	n	bias	LoA
	Cool-down/Walking phase								
T_CV_/T_PM_	50	−0.38	2.04	46	−0.68	3.36	50	−0.63	3.44
T_CV_/T_NL_	50	1.96	4.44	46	0.70	3.03	49	0.99	3.53
T_CV_/T_GM_	50	−1.02	1.63	46	−1.45	3.51	50	−1.51	3.46
T_CV_/T_SM_	50	−1.03	1.83	46	−1.71	3.69	50	−1.51	3.37
T_CV_/T_R_	50	0.16	2.70	46	−1.01	2.09	50	−0.87	2.58
	Recovery phase								
T_CV_/T_PM_	40	0.22	1.39	40	0.30	1.18	39	0.24	0.70
T_CV_/T_NL_	40	0.32	1.88	40	0.99	3.86	39	0.86	2.46
T_CV_/T_GM_	40	−0.25	1.60	40	0.06	1.70	39	0.05	1.35
T_CV_/T_SM_	40	−0.46	1.22	39	−0.33	1.59	39	−0.28	0.99
T_CV_/T_R_	40	−0.71	1.02	40	−0.64	1.15	39	−0.62	0.97

Abbreviations: W_no_ = no water application (continuously walking), W_only_ = cold water application only, W_scraping_ = cold water application and scraping, n = number of measurements, Bias = mean of differences, LoA = limits of agreement, T_CV_ = central venous temperature, T_PM_ = pectoral muscle temperature, T_NL_ = nuchal ligament temperature, T_GM_ = gluteal muscle temperature, T_SM_ = splenius muscle temperature, T_R_ = rectal temperature.

## Appendix A

**Table A1:** Exercise programs in a horse walker machine during conditioning and data collection periods (minutes).

Period	Week	Day	Speed							Total Exercise Time
			1.5 m/s		3 m/s		5.2 m/s		1.5 m/s	
Conditioning period	Week 1	M	20	→	5		→		10	35
		W	30	→	5		→		10	45
		F	40	→	10		→		10	60
	Week 2	M	40	→	15		→		10	65
		W	40	→	15	→	2	→	15	72
		F	40	→	15	→	5	→	15	75
	Week 3	M	40	→	15	→	5	→	15	75
		W	40	→	20	→	7	→	15	82
		F	40	→	20	→	10	→	15	85
Data collection period	Week 4–6		40	→	20		→		15	75

**Table A2:** Descriptive statistics of each body temperature by cool-down method and phase during the entire experimental program. (Unit: °C).

	W_no_				W_only_				W_scraping_			
	n	Mean ± SD	Min	Max	n	Mean ± SD	Min	Max	n	Mean ± SD	Min	Max
	Cool-down/Walking phase											
T_CV_	50	39.3 ± 0.5	38.2	40.3	46	38.0 ± 0.5	36.8	39.4	50	38.1 ± 0.6	36.8	39.7
T_PM_	50	39.7 ± 0.8	38.2	41.3	46	38.7 ± 1.0	36.9	40.8	50	38.7 ± 1.0	36.9	40.9
T_NL_	50	37.4 ± 1.0	35.3	39.0	46	37.3 ± 0.7	35.8	38.2	49	37.1 ± 0.8	35.5	38.3
T_GM_	50	40.3 ± 0.4	39.5	41.2	46	39.5 ± 0.8	38.1	41.1	50	39.6 ± 0.7	38.5	41.1
T_SM_	50	40.3 ± 0.6	39.2	41.8	46	39.7 ± 0.9	38.1	41.8	50	39.6 ± 0.8	38.3	41.9
T_R_	50	39.2 ± 0.4	38.1	39.9	46	39.0 ± 0.2	38.5	39.5	50	39.0 ± 0.5	38.0	39.7
	Recovery phase											
T_CV_	40	38.1 ± 0.4	37.3	39.0	40	37.7 ± 0.2	36.8	38.0	39	37.8 ± 0.4	37.1	38.5
T_PM_	40	37.9 ± 0.5	37.0	39.3	40	37.4 ± 0.3	36.7	38.0	40	37.5 ± 0.4	36.5	38.3
T_NL_	40	37.8 ± 0.4	36.7	38.9	40	36.7 ± 0.9	34.9	38.9	40	36.9 ± 0.6	35.7	37.7
T_GM_	40	38.3 ± 0.6	37.6	39.7	40	37.6 ± 0.4	37.2	39.0	40	37.7 ± 0.3	37.3	38.4
T_SM_	40	38.6 ± 0.6	37.7	39.8	39	38.0 ± 0.4	37.3	38.9	40	38.0 ± 0.4	39.3	38.9
T_R_	40	38.8 ± 0.4	38.1	39.8	40	38.3 ± 0.3	37.8	38.9	40	38.4 ± 0.4	37.6	39.1

W_no_: no water application (continuously walking), W_only_: cold water application only, W_scraping_: cold water application followed by scraping, n: number of measurements, SD: standard deviation, Min: minimum, Max: maximum, T_CV_: central venous temperature, T_PM_: pectoral muscle temperature, T_NL_: nuchal ligament temperature, T_GM_: gluteal muscle temperature, T_SM_: splenius muscle temperature, T_R_: rectal temperature, HR: heart rate.

**Table A3:** Results of Analysis of Variance (ANOVA) for measured body temperature post-exercise.

Variables	T_CV_		T_PM_		T_NL_		T_GM_		T_SM_		T_R_	
	F-Value	*p*-Value	F-Value	*p*-Value	F-Value	*p*-Value	F-Value	*p*-Value	F-Value	*p*-Value	F-Value	*p*-Value
Time	58.61	<0.01	125.15	<0.01	0.89	0.35	404.67	<0.01	210.21	<0.01	111.76	<0.01
Phase	0.36	0.55	5.28	0.02	1.28	0.26	39.77	<0.01	57.48	<0.01	0.99	0.32
Treatment	7.10	0.01	0.46	0.64	0.29	0.75	1.76	0.22	0.40	0.68	0.67	0.53
Phase:Treatment	1.13	0.35	2.11	0.12	7.13	0.01	0.88	0.42	3.81	0.02	2.66	0.07
Time:Phase	28.17	<0.01	75.81	<0.01	0.38	0.54	75.70	<0.01	45.64	<0.01	21.39	<0.01
Time:Treatment	1.13	0.35	1.89	0.15	0.27	0.75	2.54	0.08	3.10	0.05	0.94	0.39
Time:Treatment:Phase	0.59	0.56	0.51	0.60	7.46	<0.01	0.90	0.41	0.97	0.38	15.96	<0.01

Abbreviations: T_CV_ = central venous temperature, T_PM_ = pectoral muscle temperature, T_NL_ = nuchal ligament temperature, T_GM_ = gluteal muscle temperature, T_SM_ = splenius muscle temperature, T_R_ = rectal temperature. Variables: Time = time after the treadmill exercise, Phase = Cool-down/Walking phase and recovery phase, Treatment = the three different cool-down methods (no water application (continuously walking; W_no_), cold water application only (W_only_), and cold water application followed by scraping (W_scraping_).

**Table A4:** Results of estimated marginal means of linear trends analysis for measured body temperature per time measurement post-exercise (unit: °C).

	T_CV_		T_PM_		T_NL_		T_GM_		T_SM_		T_R_	
	Cooldown/ Walking	Recovery	Cooldown/ Walking	Recovery	Cooldown/ Walking	Recovery	Cooldown/ Walking	Recovery	Cooldown/ Walking	Recovery	Cooldown/ Walking	Recovery
W_no_	−0.10 ^ab^	−0.02 ^a^	−0.16 ^b^	−0.03 ^a^	+0.08 ^a^	−0.01 ^b^	−0.14 ^b^	−0.05 ^a^	−0.09 ^bc^	−0.04 ^ab^	+0.07 ^a^	−0.03 ^c^
W_only_	−0.09 ^b^	−0.01 ^a^	−0.12 ^b^	−0.02 ^a^	−0.01 ^b^	−0.00 ^b^	−0.11 ^b^	−0.04 ^a^	−0.08 ^c^	−0.02 ^a^	−0.02 ^bc^	−0.02 ^c^
W_scraping_	−0.13 ^b^	−0.01 ^a^	−0.16 ^b^	−0.02 ^a^	−0.02 ^b^	0.00 ^b^	−0.14 ^b^	−0.03 ^a^	−0.11 ^c^	−0.02 ^a^	+0.01 ^b^	−0.02 ^bc^

- Positive values (gradients) indicate increasing trends of the temperature per time measurement, while negative values indicate decreasing trends of the temperature. - Each body temperature had its own grouping letters (a, b, and c). Temperature changing trends that do not share a letter are significant differences. Abbreviations: T_CV_ = central venous temperature, T_PM_ = pectoral muscle temperature, T_NL_ = nuchal ligament temperature, T_GM_ = gluteal muscle temperature, T_SM_ = splenius muscle temperature, T_R_ = rectal temperature, W_no_ = no water application (continuously walking), W_only_ = cold water application only, W_scraping_ = cold water application followed by scraping.
